# Identification of Phytochemical-Based β-Catenin Nuclear Localization Inhibitor in NSCLC: Differential Targeting Population from Member of Isothiocyanates

**DOI:** 10.3390/molecules26020399

**Published:** 2021-01-13

**Authors:** Win Sen Heng, Shiau-Chuen Cheah

**Affiliations:** 1 Faculty of Medical Sciences, University Medical Center Groningen, Groningen 9713 GZ, The Netherlands; h.win.sen@umcg.nl; 2 Faculty of Medicine and Health Sciences, UCSI University, Kuala Lumpur 56000, Malaysia

**Keywords:** alternative medicine, natural compound, lung cancer, β-catenin nuclear localization inhibitor, alkaloid, chalcone, isothiocyanate

## Abstract

Decades of research has convinced us that phytochemical compounds contained within the plant products are the real deal, and they provide benefits such as health maintenance an d cure to illnesses. One of the deadliest noncommunicable diseases today is lung cancer, hence its disease management still deserves attention. Wnt/β-catenin pathway activation conferring cancer stem cell (CSC) activities to non-small cell lung carcinomas (NSCLCs) may explain why the disease is still difficult to cure. In the present study, we assessed several representatives of phytochemical categories consisting of alkaloids, chalcones and isothiocyanates for their inhibitory activity to nuclear localization of β-catenin—an important event for Wnt/β-catenin pathway activation, in lung cancer cell lines. Real-time cell analyzer confirmed that evodiamine (EVO), chelidonine (CHE), isoliquiritigenin (ISO), licochalcone-A (LICO), benzyl isothiocyanate (BI) and phenethylisothiocyanate (PI) exhibited anti-proliferative activities and cytotoxicities to adenocarcinoma cell line SK-LU-1 and human lung CSC primary cell line (HLCSC). Immunofluorescence assay identified that CHE, ISO, LICO, BI and PI were capable of reducing the number of cells harboring β-catenin within the nuclei of these cells. We extended the characterizations of BI and PI in Wnt-dependent squamous cell carcinoma cell line NCI-H1703 on several CSC functions and found that BI was better at inhibiting soft agar colony formation as an output of self-renewal ability, whereas PI was more effective in inhibiting the growth of multicellular tumor spheroid model mimicking micrometastases. Both however were not able to inhibit migration and invasion of NCI-H1703. In conclusion, BI could potentially be used as a safer alternative to target undifferentiated CSCs as adjuvant therapy, whereas PI could be used as chemotherapy to remove bulk tumor.

## 1. Introduction

Plants and their products have been indispensable source of living for many if not all organisms. The live support that they provide for mankind is most notably demonstrated by their use for shelter, clothing, food and health-promoting remedies. In particular, the benefits offered for health are accounted by plant secondary metabolites in foods and herbs called phytochemicals that still present as important source of drug candidates today mainly due to their highly variable chemical structures that can be used to prevent or treat illnesses, directly or indirectly [1,2].

Lung cancer is still among the deadliest noncommunicable disease and malignancy today with around two million annual new cases based on an estimation from 2018 [3]. Non-small cell lung cancer (NSCLC) hugely contributes to lung cancer’s high incidence annually at approximately 85% of total lung cancer cases. In contrast to small cell lung cancer (SCLC) subtype that only accounts for 15% of total lung cancer incidence, NSCLC, which is mainly classified as adenocarcinoma (ADC), squamous cell carcinoma (SCC) and large cell carcinoma (LCC), grows and spreads more slowly, hence has better prognosis [4]. NSCLC also has better treatment options available which include radiotherapy and surgery for early stage disease (I or II) and (neo)adjuvant chemotherapy and immunotherapy for late stage disease (III or IV) [4,5]. Some patients presenting with mutated biomarkers may benefit from the use of tyrosine kinase inhibitor (TKI) drugs [4,5]. Although immunotherapy consisting of immune-checkpoint inhibitors has made some breakthrough in recent years, some challenges remain to be addressed, including suitability, toxicity and responsiveness [6,7,8]. Nevertheless, NSCLC patients’ five-year survival rate for early, locally advanced and advanced stage of disease is still lower than 18% [4].

Tumor recurrence and metastasis are two clinical phenomena that were proposed as consequences associated to cancer stem cell (CSC) activities. Within tumor bulk, this subpopulation is therapeutically resistant and capable of reprogramming itself through epithelial-to-mesenchymal transition (EMT) into metastatic cancer cell [9]. Therefore, the clinical aim of cancer treatments is both tumor bulk eradication and CSC elimination. In NSCLC, CSC maintenance and perpetuation rely on Wnt/β-catenin pathway aberrant activation, which is mostly contributed by changes of methylation status of Wnt/β-catenin pathway negative regulators leading to pathway deregulation [10,11]. The negative regulators of Wnt/β-catenin work by preventing intracellular transducer β-catenin to localize within nucleus for Wnt target genes transcriptions [10].

Alkaloids, chalcones and isothiocyanates are among the most studied phytochemical groups in NSCLCs in the last ten years. However, their specific effect in targeting nuclear localization of β-catenin—an event that positively affects the activation of canonical Wnt pathway—is not well-studied. Moreover, the majority of the studies only focused in characterizing the most commonly used adenocarcinoma cell line A549. Hence, in the present study, we studied alkaloids represented by chelidonine (CHE) and evodiamine (EVO), chalcones represented by isoliquiritigenin (ISO) and licochalcone-A (LICO) and isothiocyanates represented by benzyl isothiocyanate (BI) and phenethyl isothiocyanate (PI) in lung cancer cells. We initially used real-time cell analyzer (RTCA) to obtain growth inhibitory profiles and IC_50_ values of the selected phytochemicals in ADC cell line SK-LU-1 and CSC primary cell line human lung CSC (HLCSC) and evaluated their inhibitory activity towards nuclear localization of β-catenin. Subsequently, we further examined phytochemicals capable of inhibiting nuclear localization of β-catenin with CSC functional assays in SCC cell line NCI-H1703. Interestingly, we found that phytochemicals belonging to the same group tend to have similar cell line-specific cytotoxic profiles with one or another displaying higher activity towards either of the tested cell lines. This difference was consistently observed when we zoomed in to see BI and PI’s effects towards CSC functions.

## 2. Results

### 2.1. The Growth Inhibitory Profiles and IC_50_ Values of Alkaloids, Chalcones and Isothiocyanates

**Figure 1:**
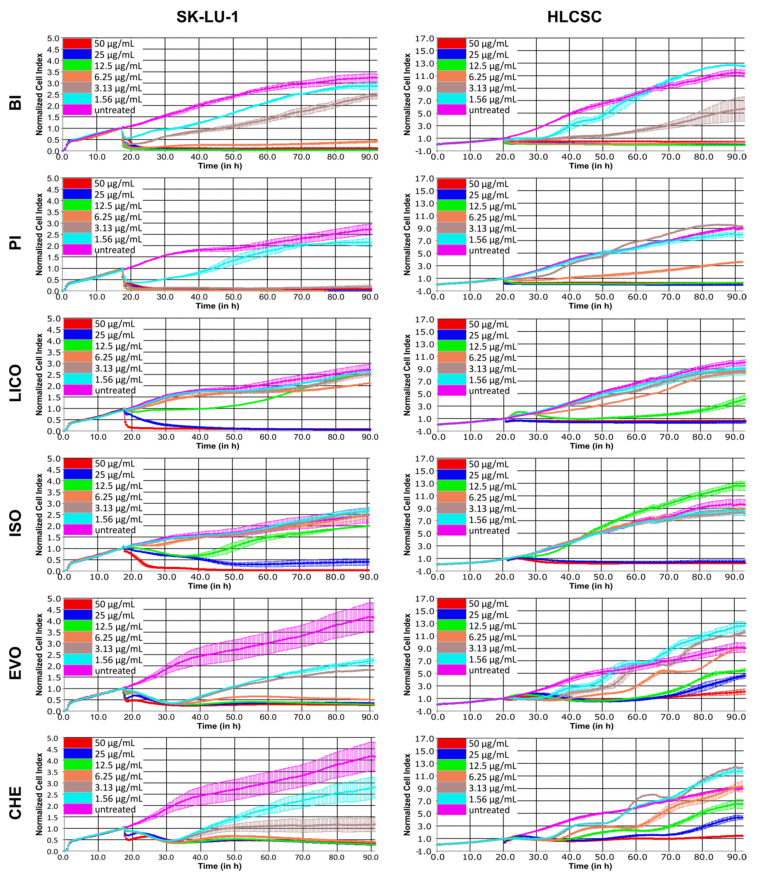
Dose–response curves depicting growth inhibitory kinetics of phytochemicals representatives of alkaloids, chalcones and isothiocyanates in SK-LU-1 and HLCSC. Various phytochemicals were administered at indicated concentrations after initial overnight growth, and the growth was continuously monitored for at least 72 h post-treatment. Curves presented are representative curves from two independent experiments. Error bars are expressed as mean ± SD from duplicated wells.

To obtain the growth inhibitory profiles of selected phytochemicals, we used real-time cell analyzer (RTCA) that measures impedance difference to assay cell attachment-dependent cell viability. The dose–response profiles are capable of revealing whether the assessed compounds exhibit anti-proliferative activity and cytotoxicity. We used SK-LU-1 as representative of differentiated ADC tumor cell line and HLCSC as representative of undifferentiated CSC cell line. HLCSC was characterized by endorsing company to enrich with CSC properties, including markers expressions (CD133, aldehyde dehydrogenase (ALDH), stage-specific embryonic antigen 3/4 (SSEA3/4), alkaline phosphatase, octamer-binding transcription factor 4 (OCT4) and CD43) and in vivo tumorigenicity (< 1000 cells). Figure 1 shows the growth inhibitory profiles of representatives of alkaloid, chalcone and isothiocyanate in SK-LU-1 and HLCSC. Phytochemicals exhibited dose-dependent cytotoxicity and anti-proliferative activity which were most consistently seen at ≤6.25 μg/mL treatments. A two-way repeated measures ANOVA was used to statistically analyze the effects of time, treatment concentrations and the interactions of those two variables towards the growth of SK-LU-1 and HLCSC. Table 1 shows the summary of the analyses. Treatments variable always had the largest effect size towards response difference displayed in the curves, except for alkaloids treatments in HLCSC, in which time had a larger effect size than the other variables. Phytochemicals in the same category noticeably have more similar growth inhibitory profiles in a cell line-specific manner. For instance, both EVO and CHE had early cytotoxicity (negative gradient) and late growth arrest (flat curve) in SK-LU-1 at all tested concentrations, except 1.56 and 3.13 μg/mL treatments for which some recovery (positive gradient) was seen (*p* < 0.001). In HLCSC, EVO and CHE similarly induced multiple growth arrest occasions transitioning in between recoveries (*p* < 0.001); however, at least two tested concentrations (1.56 and 3.13 μg/mL) of EVO and CHE did not evoke significant reduction of growth (*p* > 0.05). BI and PI were highly cytotoxic, killing SK-LU-1 and HLCSC at an early time-point at treatment concentration of ≥ 6.25 μg/mL (*p* < 0.001). They were also anti-proliferative towards HLCSC at early time-point of 1.56 and 3.13 μg/mL treatments. As expected, PI did not significantly induce HLCSC cell killing at these concentrations, whereas BI’s was only unable to reduce significant growth at 1.56 μg/mL treatment (*p* > 0.05). LICO and ISO are the least similar group and two of the least effective phytochemicals tested. They induced a complete cell killing in SK-LU-1 and HLCSC at 25 and 50 μg/mL treatments (only at 50 μg/mL for ISO in SK-LU-1; *p* < 0.001). In LICO- and ISO-treated SK-LU-1, at least three concentrations (1.56, 3.13 and 6.25 μg/mL) failed to induce significant inhibition, whereas this occurred in HLCSC for at least the two lowest mentioned concentrations (*p* > 0.05). None of the phytochemical compounds tested was as effective as positive control compound SAL that induced either cytotoxicity or growth arrest in SK-LU-1 and HLCSC at all treatment concentrations, except in 1.56 μg/mL-treated SK-LU-1 (Figure S1; *p* < 0.001).

The growth inhibitory effectiveness of phytochemicals was also determined by evaluating IC_50_ values from specific time-intervals of dose–response curves. Table 2 summarizes the IC_50_ values of respective phytochemicals in SK-LU-1 and HLCSC at 24, 48 and 72 h post-treatment. As similarly shown by the dose–response curve, the IC_50_ values indicate that SAL was more superior than all tested phytochemicals with especially greater potency towards HLCSC. PI, EVO and CHE showed similar activity as SAL towards SK-LU-1 cells (8–19 μM), but none was as effective as SAL against HLCSC. It is worth noting that, from each category of phytochemicals, BI, LICO and EVO were the members which were relatively more potent in inhibiting HLCSC’s growth in isothiocyanate, chalcone and alkaloid, respectively.

### 2.2. Chalcones and Isothiocyanates Modulated Reduction of β-Catenin Nuclear Localization

**Figure 2:**
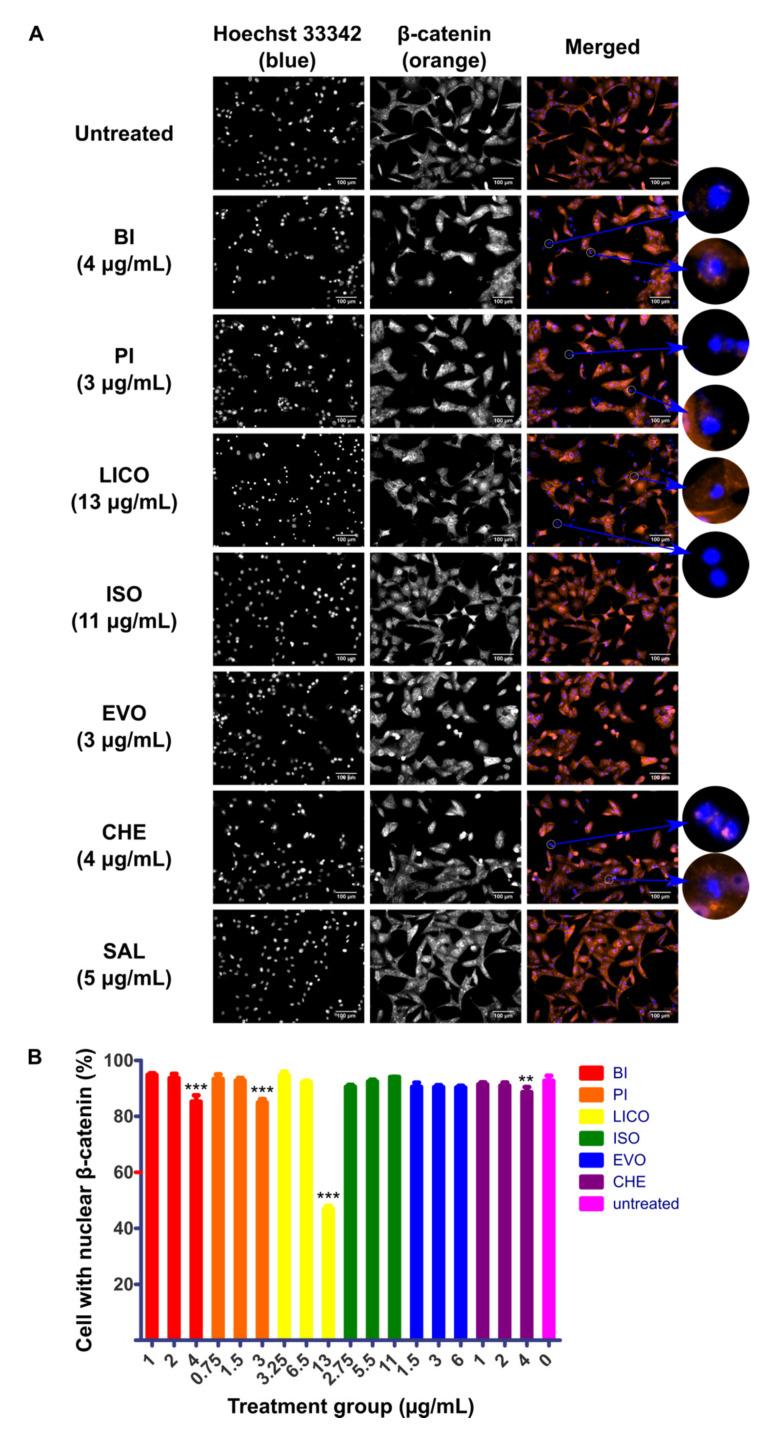
Immunofluorescence detecting localization behavior of β-catenin in phytochemicals-treated SK-LU-1. SK-LU-1 was pre-treated with GSK3i at 8 μM for 24 h followed by phytochemicals treatments for 24 h. (**A**) Representative micrographs depicting immunofluorescence staining of β-catenin (orange) counterstained with Hoechst 33342 (blue) to indicate nuclei in phytochemicals-treated SK-LU-1. White circles highlight cells with diminished β-catenin localization in nucleus. Scale bars represent 100 μm. (**B**) Graph representing percentage of SK-LU-1 with nuclear β-catenin calculated in response to treatment at ¼ IC_50_, ½ IC_50_ and IC_50_ concentrations of respective phytochemicals. The quantification data were derived from a representative immunostaining experiment. Error bars are expressed as mean ± SD from triplicate data in an independent experiment. Statistical significance is expressed as *** *p* < 0.001; ** *p* < 0.01.

**Figure 3:**
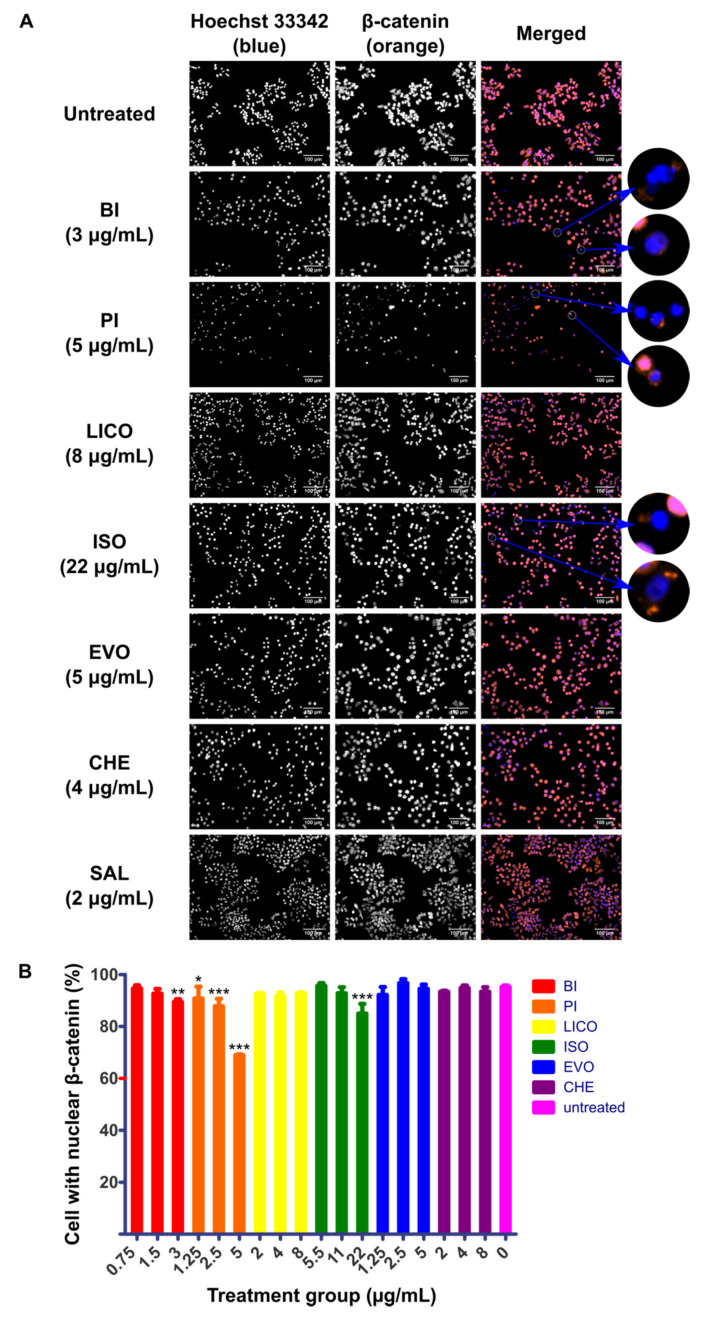
Immunofluorescence detecting localization behavior of β-catenin in phytochemicals-treated HLCSC. HLCSC was pre-treated with GSK3i at 8 μM for 24 h followed by phytochemicals treatments for 24 h. (**A**) Representative micrographs depicting immunofluorescence staining of β-catenin (orange) counterstained with Hoechst 33342 (blue) to indicate nuclei in phytochemicals-treated HLCSC. White circles highlight cells with diminished β-catenin localization in nucleus. Scale bars represent 100 μm. (**B**) Graph representing percentage of HLCSC with nuclear β-catenin calculated in response to treatment at ¼ IC_50_, ½ IC_50_ and IC_50_ concentrations of respective phytochemicals. The quantification data were derived from a representative immunostaining experiment. Error bars are expressed as mean ± SD from triplicate data in an independent experiment. Statistical significance is expressed as *** *p* < 0.001; ** *p* < 0.01; * *p* < 0.05.

The nuclear localization of β-catenin is considered to be the hallmark activation event of Wnt/β-catenin pathway and therefore inhibition of this event could potentially limit downstream pro-tumorigenic effect of the pathway. In addition to the cytotoxicity and growth arrest capability of phytochemicals that we have shown above, we were interested to characterize the ability of these phytochemicals to disrupt the activity of Wnt/β-catenin signaling by visualizing the nuclear localization activity in response to phytochemicals treatment. SK-LU-1 and HLCSC were first pre-treated with GSK3i at 8 μM for 24 h to maximize the nuclear localization of β-catenin before administering phytochemicals for another 24 h. At least three phytochemicals in each cell lines tested could inhibit localization activity of β-catenin indicated by increased number of cells that only stained blue for nuclei, regardless of whether the cytoplasm stained orange for β-catenin. In SK-LU-1, BI (4 μg/mL, *p* < 0.001), PI (3 μg/mL, *p* < 0.001), LICO (13 μg/mL, *p* < 0.001) and CHE (4 μg/mL, *p* < 0.01) significantly inhibited the localization of β-catenin to nucleus (Figure 2A,B). On the other hand, BI (3 μg/mL, *p* < 0.01), PI (1.25 μg/mL, *p* < 0.05; 2.5 μg/mL, *p* < 0.001; 5 μg/mL, *p* < 0.001) and ISO (22 μg/mL, *p* < 0.001) inhibited the localization in HLCSC (Figure 3A,B). All of these indicate that isothiocyanates (BI and PI) and chalcones (ISO and LICO) may be potent inhibitors of β-catenin nuclear localization as either one or both of the respective phytochemical members exhibited their effects in both examined cell lines. Due to their wider applicability and lower IC_50_ values to inhibit β-catenin nuclear localization, we selected BI and PI for further characterizations.

### 2.3. Both Isothiocyanate Members were Equally Potent to Inhibit the Growth of Wnt-Dependent Cell Line NCI-H1703 Although with Differing Sensitivity in Inhibiting Undifferentiated and Differentiated Lung Cancer Cells

To focus observations on canonical Wnt-dependent growth, we used NCI-H1703 for downstream experiments. NCI-H1703 was previously identified to depend its survival on the expressions of WNT1 and WNT2 ligands [12,13]. Furthermore, NCI-H1703 is a lung SCC cell line, therefore characterization in this cell line can add to our observation in another lung carcinoma histological subtype for the selected phytochemicals. 

Growth inhibitory profiles of BI and PI were similarly obtained in NCI-H1703 by using RTCA. The dose–response curves indicate that the isothiocyanate compounds could dose-dependently inhibit NCI-H1703 (Figure S2). Based on the curve, we determined the IC_50_ values and used them to extrapolate IC_20_ and IC_80_ values. These values enabled us to see phytochemicals relative inhibitory effectiveness among different cell lines. Table 3, Table 4, Table 5 show IC_20_, IC_50_ and IC_80_ values of BI, PI and reference compound SAL, respectively, for the evaluation of inhibitory effectiveness in NCI-H1703, SK-LU-1 and HLCSC. NCI-H1703 shows similar sensitivity to BI and PI throughout the three measured time-points across three different IC values. This is judged relatively to other tested cell lines consisting of SK-LU-1 and HLCSC. Table 3 shows that NCI-H1703 and HLCSC were relatively more sensitive to BI compared to SK-LU-1, whereas Table 4 demonstrates that NCI-H1703 and SK-LU-1 were relatively more sensitive to PI, but HLCSC was relatively more resistant to it. As expected, only HLCSC consistently showed higher sensitivity to SAL (Table 5). All of these suggest that BI and PI could more effectively target either undifferentiated (e.g., HLCSC) or differentiated (e.g., SK-LU-1) lung cancer cells, but both could be equally potent in inhibiting the growth of Wnt-dependent cells NCI-H1703.

### 2.4. BI and PI Inhibited the Growth of NCI-H1703’s Soft Agar Colony and Spheroid, But not Migration and Invasion

**Figure 4:**
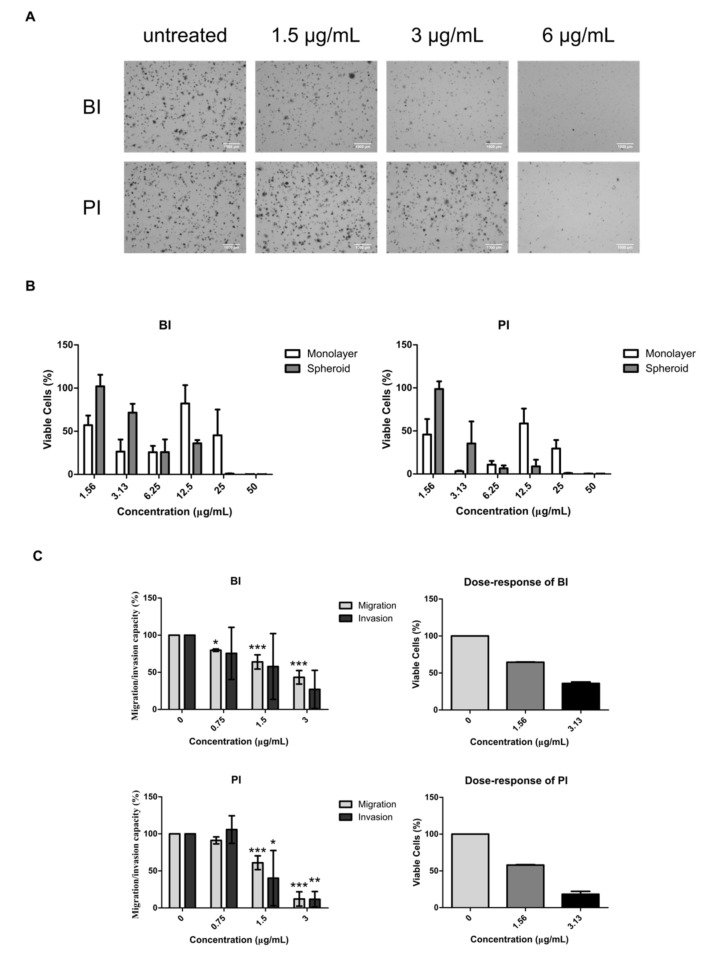
Soft agar colony formation, multicellular tumor spheroid growth, migration and invasion in BI- and PI-treated NCI-H1703. (**A**) Representatives micrographs of colony formation of NCI-H1703 in soft agar after BI and PI treatment at indicated concentrations. Scale bars represent 1000 μm. (**B**) Graphs presenting cell viability of parallel set-up of monolayer and spheroid models that were treated with BI and PI at indicated concentrations. (**C**) Graphs presenting migration and invasion of NCI-H1703 (left) after BI and PI treatments and dose–response graphs (right) of respective phytochemicals at indicated concentrations. Error bars in spheroid cell viability, migration and invasion data were expressed as mean ± SD from three independent experiments. Error bars in dose–response of viable cells data were expressed as mean ± SD from two independent experiments. Statistical significance is expressed as *** *p* < 0.001; ** *p* < 0.01; * *p* < 0.05.

Wnt/β-catenin signaling is associated with increased CSC functions that include proliferation, survival and motility. To determine whether BI and PI’s difference in inhibiting differentiated and undifferentiated lung cancer cells also affects their abilities in inhibiting these CSC functions, we used NCI-H1703 cell line to evaluate both phytochemicals inhibitory activities towards CSC properties such as soft agar colony formation, spheroid growth, migration and invasion. Both isothiocyanate compounds were observed to exhibit similar inhibitory effect towards NCI-H1703’s growth and nuclear localization of β-catenin at IC_50_ concentrations. Hence, we picked IC_50_ values, as well as concentrations two-fold lower and two-fold higher than IC_50_ values, for soft agar colony treatments. Figure 4 shows inhibitions of BI and PI towards the aforementioned CSC properties. Long-term colony formation that relies on the viability of CSC was more effectively inhibited by BI at 1.5 and 3 μg/mL compared to PI where no inhibition was observed at the same concentrations (Figure 4A). On the other hand, PI was more potent in inhibiting the growth of multicellular tumor spheroid (MCTS) model of NCI-H1703 in dose-dependent manner (Figure 4B). Spheroids treated with increasing concentrations of BI and PI may appear as compact structure, which indicate intact and viable structure, whereas dying spheroids are morphologically loose, hence appear bigger in size (Figure S3). Both BI and PI induced a loose appearance to spheroids when treated with ≥ 6.25 μg/mL as also depicted in Figure 4B with their low cell viability. Figure 4C depicts migration and invasion capability of NCI-H1703 in response to BI and PI treatments. Sixteen hours after the onset of treatments, there was significant reduction of percentage of migration and invasion’s CI in BI- and PI-treated NCI-H1703 relative to control. However, dose–response of BI and PI at the concentrations that had significant reduction of migration and invasion signal suggested that the reduction solely was due to reduction of cell viability. Therefore, the measured activity was an output of cytotoxicity, but not migration and invasion activity decrement.

## 3. Discussion

In the present study, we examined dietary and herbal phytochemicals for their inhibitory effects in NSCLCs. Among phytochemicals representatives of alkaloids comprising of CHE and EVO, chalcones comprising of ISO and LICO and isothiocyanates comprising of BI and PI, we found that members of isothiocyanates exhibit mutual inhibitory activity towards the activation of Wnt/β-catenin pathway, but further characterization showed different targeting preference between undifferentiated lung CSC (HLCSC) and differentiated lung cancer cells (SK-LU-1). By using SAL as positive control compound against CSCs on the basis of IC_20_, IC_50_ and IC_80_ values, we confirmed that BI was more potent against HLCSC compared to SK-LU-1, whereas PI was more desirable to inhibit SK-LU-1. Interestingly, these patterns were replicated in assays such as soft agar colony formation assay where BI was seen to be more effective in inhibiting colony growth and MCTS assay where PI reduced the number of viable cells more effectively.

Wnt/β-catenin pathway is a conserved embryonic signaling that is still actively used by adult lung tissue. For instance, alveolar epithelial cells (AECs) II can constitutively activate canonical Wnt pathway to maintain stemness with Wnt stimulation from adjacent fibroblast cells and they can switch on Wnt-autocrine signaling during severe injury [14]. Wnt-fibroblast growth factor (FGF) signaling is utilized by mesenchymal cells in the airway to support epithelial cells during injury [15]. NSCLCs in respective tissue origins (i.e., SCC from the airway and ADC from air sacs) use the same Wnt/β-catenin mechanism, but with some deregulations such as through aberrant inhibition or decreased expression of Wnt negative regulators, and increased expression of Wnt ligands or transducers such as Dishevelled (DVL) or β-catenin [10,11,16]. Catenin B1 gene (*CTNNB1*) may be mutated in ADC leading to evasion of proteosomal degradation and constitutive activation of Wnt pathway [17]. Cells with increased nuclear accumulation of β-catenin, for example, enrich for CSC traits such as higher expression of nuclear Nanog in NSCLCs, which predicts for poor prognosis of NSCLC [18]. CSC enrichment or inability to kill CSC population was deemed to be the root of therapeutic resistance problem in clinics today, including for NSCLCs [9].

Hence, in the present study, we looked at potential phytochemicals which are able to diminish localization of β-catenin to nucleus and found that chalcone and isothiocyanate phytochemicals were able to decrease this hallmark activation event. Interestingly, other chalcone and isothiocyanate members have also been found to inhibit Wnt/β-catenin pathway. The chalcone cardamonin has been demonstrated to reduce nuclear β-catenin localization in triple negative breast cancer cell line BT-549 [19]. Other chalcones including derricin and derricidin have also been reported to possess such effect when treated to HCT116 colon cancer cell line [20]. An isothiocyanate compound, sulforaphane, was reported to inhibit canonical Wnt transcriptional activity in two separate studies, as demonstrated by TCF/LEF reporter assays in breast CSCs and colorectal cancer cell lines SW480, DLD1 and HCT116 [21,22]. In this study, while isothiocyanate members consistently exhibited this effect, chalcones did not. It is not clear what caused the inconsistency, but concentration-specific targeting may result in concentration-independent dose–response. Our previous study on chelerythrine chloride identified this specific-targeting dose-independent effect of nuclear localization of β-catenin in NCI-H1703 and SK-LU-1 [23]. At low concentration, the effect was higher. In this case, inhibitory effect towards nuclear localization of β-catenin for ISO in SK-LU-1 and LICO in HLCSC may be exhibited at either lower or higher concentration than those tested in this study. Future study is warranted to confirm how widespread the dose-independent specific-targeting phenomenon is.

The most important CSC function which confers on CSC the ability to perpetuate and maintain tumor is the self-renewal ability [24]. This ability can be demonstrated through soft agar colony-forming assay in vitro. Only CSC possessing self-renewal ability is capable of forming visible long-term colony in soft agar culturing under an anchorage independence situation [24]. On the other hand, MCTS is a three-dimensional (3D) tumor model that mimics the structure of avascular micrometastases in vivo [25]. Besides that, its multilayered cells architecture also simulates the in vivo physiological condition of tumor bulk that is often presented with complex relationships among chemical gradients, inner hypoxia, cell–cell interactions and cell–matrix interactions that ultimately affect drug response [26]. In addition, due to its layering barrier by differentiated cells, drug penetration is an important factor considered when using this drug testing model since it may provide physical protective-barrier for CSC that may lie inside [27]. As such, BI at 1.5 and 3 μg/mL may be specifically targeting CSCs leading to lower capability of NCI-H1703 cells to form larger colonies in higher density. PI may majorly rely on its differentiated cells cytotoxicity to decrease the number of viable cells in NCI-H1703 MCTS model that mainly consist of differentiated cells maintained by small number of CSCs. Penetration may not be a differing factor since BI and PI have approximately the same molecular size (149.21 vs. 163.24 g/mol, respectively).

To assess the selectivity of phytochemicals towards cancer growth inhibitions, we performed dose–response assessment of phytochemicals in normal lung fibroblast cell line IMR-90. However, the results suggest that IMR-90 cells were as susceptible as other lung cancer cell lines used in the present study (Figure S4). Although we did not confirm the selectivity of growth inhibitory effect towards cancer cells, phytochemicals assessed in this study have been widely incorporated in products used for daily life. LICO and ISO are compounds isolated from licorice, a natural sweetener ingredient for some snacks and drinks [28]. Isothiocyanates can be obtained from cruciferous vegetables such as cabbage, broccoli and cauliflower [29]. EVO is found in evodia fruits commonly used as traditional medicine in China [30]. CHE is the major alkaloid derived from the *Chelidonium majus* medicinal plant used in Europe and China [31]. With all these histories of consumptions by human, the described phytochemicals are undoubtedly safe at low concentration. Besides that, we noticed a therapeutic window for selective CSC inhibition whereby at a concentration that modulated reduction of colony formation, BI was not cytotoxic to the 3D model that better replicates multicellular conformation of tumor bulk—a model that perhaps also better mimics the architecture of normal epithelial cells in vivo when compared to monolayer 2D model.

## 4. Materials and Methods 

### 4.1. Cell Culture

Human lung ADC cell line SK-LU-1 and human lung SCC cell line NCI-H1703 were purchased from American Type Culture Collections (ATCC, Manassas, VA, USA). Human lung cancer stem cell (HLCSC) primary cell line was purchased from Celprogen (Torrance, CA, USA). SK-LU-1 and NCI-H1703 were routinely maintained in Gibco’s Dulbecco’s modified Eagle’s medium (DMEM) with 4.5 g/L d-glucose (Thermo Fisher Scientific, Waltham, MA, USA) supplemented with 1% (*v*/*v*) of 100 mM Gibco’s sodium pyruvate, 1% (*v*/*v*) of 10,000 U/mL Penicillin–10,000 μg/mL Streptomycin (Pen-Strep) and 10% (*v*/*v*) of Gibco’s fetal bovine serum (FBS). HLCSC was routinely maintained in human lung cancer stem cell complete growth medium with serum (Celprogen). All cell cultures were maintained in 5% CO_2_ humidified atmospheric condition CO_2_ incubator at 37 °C. Passaging was regularly performed with Sigma-Aldrich’s (St. Louis, MO, USA) 0.25% (*w*/*v*) trypsin–ethylenediaminetetraacetic acid (EDTA) solution.

### 4.2. Chemicals

Benzyl isothiocyanate (BI) and licochalcone-A (LICO) were obtained from Sigma-Aldrich. Chelidonine (CHE), evodiamine (EVO), isoliquiritigenin (ISO) and phenethyl isothiocyanate (PI) were obtained from Chromadex (Los Angeles, CA, USA). Salinomycin (SAL) was obtained from AdooQ Bioscience (Irvine, CA, USA). Glycogen Synthase Kinase-3 inhibitor X (GSK3i) was purchased from Calbiochem (San Diego, CA, USA). All phytochemicals were prepared by dissolving in dimethyl sulfoxide (DMSO, Sigma-Aldrich) at 10 mg/mL stock.

### 4.3. Cell Viability, Cytotoxicity and Anti-Proliferative Assay

Real time cell analyzer (RTCA) was performed as described previously using an E-plate 16 on xCELLigence RTCA-Dual Purpose (DP) platform (ACEA Bioscience, San Diego, CA, USA) [23]. Briefly, 50 μL of complete medium ere added into each well for 30-min incubation before acquiring background measurements. Optimized cell density (1 × 10^4^ cells/well for SK-LU-1 and 1.25 × 10^4^ cells/well for HLCSC and NCI-H1703) was seeded upon background measurement and was incubated for 30 min at room temperature in the biological safety cabinet in order to facilitate cells settling before putting back onto the RTCA-DP platform for overnight incubation. Subsequently, phytochemicals were added to each well and incubated at various concentrations for up to 72 h. RTCA software 2.0 was used to generate kinetic dose–response curve by normalizing the cell index (CI) to the time when the phytochemical was added. IC_50_ values were estimated by plotting CI against concentration using algorithm for sigmoidal dose–response curve with variable slope.

CellTiter-Glo 3D cell viability assay (Promega, Madison, WI, USA) was used to evaluate the growth of spheroid culture (refer to spheroid generation section) along with the parallel run of monolayer culture (seeded at 1 × 10^4^ cells/well). After 24 h treatment with selected phytochemicals at indicated concentrations, each of the spheroids was transferred to a single well of an opaque-white plate for cell viability measurement according to the manufacturer’s protocol. Monolayer culture was directly seeded in the opaque-white plate. Luminescence was measured as relative luminescence unit (RLU) by using FLUOstar Omega microplate reader (BMG LABTECH, Ortenberg, Germany). Each sample was normalized against respective vehicle control. GraphPad Prism v5.01 software (San Diego, CA, USA) was used to plot concentration against percentage of viability.

### 4.4. Immunocytochemistry

Immunofluorescence assay was performed as described previously [23]. SK-LU-1 and HLCSC were first treated with GSK3i for 24 h upon growing overnight in order to optimally increase β-catenin localization before the onset of phytochemicals treatments. Immunofluorescence was performed by using Cellomics^®^ Beta-Catenin Activation Kits (Thermo Scientific, Waltham, MA, USA) in accordance with the manufacturer’s protocol. Five random, non-overlapping frames were captured from each well by using Axio Vert.A1 inverted microscope equipped with HXP-120V light source and Axiocam MR R3 camera (Carl Zeiss, Oberkochen, Germany). Cells harboring nuclear β-catenin were manually counted by using ImageJ’s cell counter plugin and the positive counts were divided by the respective total number of cells in the captured frames to obtain percentage of cells with positive nuclear β-catenin [32]. Treated samples were compared with untreated Wnt-activated negative control.

### 4.5. Soft Agar Colony Formation Assay

The assay was performed as described previously with some modifications [23,33]. Briefly, top/bottom agar was established at 0.35%/0.6% (*w*/*v*) with noble agar (Becton Dickinson, Franklin Lakes, NJ, USA) in a six-well plate. NCI-H1703 cells were suspended within the top agar at 5 × 10^4^ cells. Treated and untreated groups were kept wet with either medium or medium containing selected phytochemicals at adjusted concentrations by renewal of every three days. After three weeks, the plate was collected and stained with 0.01% (*v*/*v*) of crystal violet for 30 min at room temperature. The plate was then washed for three times with PBS. SZ51 zoom stereo microscope (Olympus, Shinjuku, Tokyo, Japan) was used to visualize the colonies and Toupcam microscope digital camera (ToupTek, Zhejiang, China) equipped with Toupview software (ToupTek, Zhejiang, China) was used to capture the micrographs.

### 4.6. Multicellular Tumor Spheroid Generation Assay

NCI-H1703 was seeded at 400 cells/well in a Costar ultra-low attachment microplate (Corning Inc., Corning, NY, USA) by using the same DMEM medium used for monolayer culture. After an overnight incubation, Matrigel (final concentration at 2.5% (*v*/*v*), Corning Inc.) was added gently towards the center of the well to facilitate compact formation of spherical structure. The spheroid was optimized to grow to 300–400 μm in diameter four days post-generation. The diameter of spheroid was measured by using Zen 2 pro software (Carl Zeiss).

### 4.7. Migration and Invasion Assay

RTCA-DP system was similarly used for migration and invasion evaluation. CIM-plate 16 that consists of two chambers was used for this purpose. Overnight serum-starved 4 × 10^4^ NCI-H1703 cells were seeded with serum-free medium at the upper chamber upon background measurement, whereas medium containing 10% (*v*/*v*) of FBS was added to the lower chamber to serve as chemoattractant. Serum-free medium in the lower chamber serves as control. For migration, no Matrigel coating is necessary, but, for invasion, 1:30 Matrigel was used to coat upper chamber 4 h prior to cell seeding. Selected phytochemicals were added at indicated concentrations upon seeding. The migration–invasion was monitored for up to 24 h. RTCA software 2.0 was used to generate dose–response kinetic curve of both migration and invasion of NCI-H1703 by expressing CI against time.

### 4.8. Statistical Analysis

All data were expressed as mean ± standard deviation (SD). Statistical significance was evaluated by using one-way analysis of variance (ANOVA) in SPSS 22.0 software (IBM SPSS, Chicago, IL, USA). Dunnett’s multiple comparison test was used for Post-hoc. The effect of time and treatment concentrations as well as the interactions of both towards cell growth in dose–response curve was statistically analyzed by using two-way repeated measures ANOVA in GraphPad Prism v5.01 software. Simple main effect analysis of treatment concentrations was analyzed by using Bonferroni test. Mean difference for a particular treatment concentration was considered significant if the majority observed time-points had statistical significance when compared to those of untreated control. Statistical significance was expressed as *** *p* < 0.001; ** *p* < 0.01; * *p* < 0.05. All data were collected from three independent experiments unless otherwise specified.

## 5. Conclusions

Through our characterizations, we confirmed that alkaloids EVO and CHE, chalcones LICO and ISO and isothiocyanates BI and PI exhibited decent anti-proliferative and cytotoxic effects. These effects may in part be due to inhibition towards β-catenin nuclear localization such as those observed in chalcones and isothiocyanates treatments. Our study and literature search suggest that the chalcones and isothiocyanates’ Wnt/β-catenin inhibitory activity may be something generalizable for their classes since other members of chalcone and isothiocyanate were also found to have similar effect. Further characterizations interestingly revealed that isothiocyanate members BI and PI had different potency in targeting different cellular components judging from the sensitivity of undifferentiated and differentiated lung cancer cells in response to the treatments, even though they only differ by an extra methylene group in PI’s chemical structure. This hidden effect was further proven in the soft agar CSC assay, where CSC is enriched, and the MCTS model, where CSC is present in minute number. As constituent phytochemicals that are present in dietary vegetables, BI and PI may offer an alternative for cancer prevention. Therapeutically, BI and PI combinations or BI used as adjuvant therapy with chemotherapy may be seen as ideal composite therapy for CSC eradication and tumor bulk clearance. Future study may need to assess their potential use further in clinics as β-catenin nuclear localization inhibitors.

## Figures and Tables

**Table 1:** Summary of the two-way repeated measures ANOVA analysis results of alkaloids, chalcones and isothiocyanates in SK-LU-1 and HLCSC.

Phytochemical Category	Phytochemical Compound	Cell Line	Independent Variables	Results
Isothiocyanate	BI	SK-LU-1	Time	F (184, 1288) = 992.6, *p* < 0.0001, R^2^ = 0.08
			Treatment	F (6, 1288) = 1095, *p* < 0.0001, R^2^ = 0.79
			Interaction	F (1104, 1288) = 250.8, *p* < 0.0001, R^2^ = 0.13
		HLCSC	Time	F (184, 1288) = 352.5, *p* < 0.0001, R^2^ = 0.12
			Treatment	F (6, 1288) = 301.2, *p* < 0.0001, R^2^ = 0.65
			Interaction	F (1104, 1288) = 105.6, *p* < 0.0001, R^2^ = 0.22
	PI	SK-LU-1	Time	F (184, 1288) = 195.1, *p* < 0.0001, R^2^ = 0.03
			Treatment	F (6, 1288) = 1584, *p* < 0.0001, R^2^ = 0.85
			Interaction	F (1104, 1288) = 116.9, *p* < 0.0001, R^2^ = 0.12
		HLCSC	Time	F (184, 1288) = 4931, *p* < 0.0001, R^2^ = 0.16
			Treatment	F (6, 1288) = 777.7, *p* < 0.0001, R^2^ = 0.67
			Interaction	F (1104, 1288) = 851.8, *p* < 0.0001, R^2^ = 0.17
Chalcone	LICO	SK-LU-1	Time	F (184, 1288) = 362.4, *p* < 0.0001, R^2^ = 0.11
			Treatment	F (6, 1288) = 180.9, *p* < 0.0001, R^2^ = 0.78
			Interaction	F (1104, 1288) = 56.6, *p* < 0.0001, R^2^ = 0.10
		HLCSC	Time	F (184, 1288) = 1362, *p* < 0.0001, R^2^ = 0.27
			Treatment	F (6, 1288) = 201, *p* < 0.0001, R^2^ = 0.55
			Interaction	F (1104, 1288) = 149.5, *p* < 0.0001, R^2^ = 0.18
	ISO	SK-LU-1	Time	F (184, 1288) = 365.3, *p* < 0.0001, R^2^ = 0.12
			Treatment	F (6, 1288) = 307.5, *p* < 0.0001, R^2^ = 0.75
			Interaction	F (1104, 1288) = 62, *p* < 0.0001, R^2^ = 0.13
		HLCSC	Time	F (184, 1288) = 1985, *p* < 0.0001, R^2^ = 0.31
			Treatment	F (6, 1288) = 362.8, *p* < 0.0001, R^2^ = 0.52
			Interaction	F (1104, 1288) = 175.1, *p* < 0.0001, R^2^ = 0.17
Alkaloid	EVO	SK-LU-1	Time	F (184, 1288) = 151.7, *p* < 0.0001, R^2^ = 0.08
			Treatment	F (6, 1288) = 52.7, *p* < 0.0001, R^2^ = 0.76
			Interaction	F (1104, 1288) = 47.9, *p* < 0.0001, R^2^ = 0.15
		HLCSC	Time	F (184, 1288) = 1929, *p* < 0.0001, R^2^ = 0.48
			Treatment	F (6, 1288) = 206.3, *p* < 0.0001, R^2^ = 0.33
			Interaction	F (1104, 1288) = 130.5, *p* < 0.0001, R^2^ = 0.19
	CHE	SK-LU-1	Time	F (184, 1288) = 59.4, *p* < 0.0001, R^2^ = 0.06
			Treatment	F (6, 1288) = 38.9, *p* < 0.0001, R^2^ = 0.74
			Interaction	F (1104, 1288) = 31.4, *p* < 0.0001, R^2^ = 0.18
		HLCSC	Time	F (184, 1288) = 328.3, *p* < 0.0001, R^2^ = 0.49
			Treatment	F (6, 1288) = 121.8, *p* < 0.0001, R^2^ = 0.34
			Interaction	F (1104, 1288) = 17.2, *p* < 0.0001, R^2^ = 0.16
Positive control	SAL	SK-LU-1	Time	F (184, 1288) = 71.5, *p* < 0.0001, R^2^ = 0.04
			Treatment	F (6, 1288) = 80.9, *p* < 0.0001, R^2^ = 0.79
			Interaction	F (1104, 1288) = 47.1, *p* < 0.0001, R^2^ = 0.16
		HLCSC	Time	F (184, 1288) = 933.5, *p* < 0.0001, R^2^ = 0.15
			Treatment	F (6, 1288) = 591.4, *p* < 0.0001, R^2^ = 0.65
			Interaction	F (1104, 1288) = 205.4, *p* < 0.0001, R^2^ = 0.20

**Table 2:** IC_50_ values of alkaloids, chalcones and isothiocyanates in SK-LU-1 and HLCSC.

IC50 Values (mean ± SD)							
Phytochemical Category	Phytochemical Compound	SK-LU-1					
		24 h		48 h		72 h	
		μg/mL	μM	μg/mL	μM	μg/mL	μM
Isothiocyanate	BI	3.96 ± 0.27	26.54 ± 1.80	4.54 ± 1.35	30.39 ± 9.05	4.99 ± 1.04	33.41 ± 6.97
	PI	2.68 ± 0.01	16.39 ± 0.04	2.69 ± 0.00	16.48 ± 0.00	2.81 ± 0.04	17.18 ± 0.22
Chalcone	LICO	12.75 ± 0.38	37.68 ± 1.13	13.96 ± 1.41	41.24 ± 4.16	16.28 ± 1.79	48.09 ± 5.29
	ISO	10.93 ± 2.28	42.63 ± 8.91	16.73 ± 0.40	65.29 ± 1.55	17.11 ± 0.79	66.75 ± 3.06
Alkaloid	EVO	5.71 ± 0.69	18.81 ± 2.26	5.41 ± 1.06	17.83 ± 3.50	4.33 ± 0.36	14.26 ± 1.19
	CHE	4.32 ± 0.21	12.21 ± 0.58	2.86 ± 0.55	8.09 ± 1.56	2.74 ± 0.47	7.74 ± 1.34
Positive control	SAL	10.40 ± 2.33	13.84 ± 3.10	9.96 ± 1.10	13.26 ± 1.46	10.18 ± 0.33	13.56 ± 0.43
**Phytochemical Category**	**Phytochemical Compound**	**HLCSC**					
		**24 h**		**48 h**		**72 h**	
		**μg/mL**	**μM**	**μg/mL**	**μM**	**μg/mL**	**μM**
Isothiocyanate	BI	2.88 ± 0.24	19.30 ± 1.61	2.80 ± 0.11	18.73 ± 0.71	3.14 ± 0.23	21.04 ± 1.52
	PI	5.14 ± 0.20	31.49 ± 1.21	6.10 ± 0.07	37.37 ± 0.43	6.27 ± 0.16	38.38 ± 1.00
Chalcone	LICO	8.27 ± 1.49	24.42 ± 4.41	8.65 ± 0.63	25.55 ± 1.86	12.01 ± 0.59	35.49 ± 1.76
	ISO	22.25 ± 0.67	86.81 ± 2.62	23.01 ± 0.12	89.76 ± 0.47	23.52 ± 0.45	91.77 ± 1.74
Alkaloid	EVO	4.71 ± 1.49	15.53 ± 4.90	7.72 ± 0.72	25.45 ± 2.38	9.13 ± 0.54	30.10 ± 1.77
	CHE	8.2 ± 0.44	23.21 ± 1.24	7.86 ± 0.04	22.23 ± 0.10	12.57 ± 2.72	35.57 ± 7.68
Positive control	SAL	3.46 ± 0.35	4.61 ± 0.47	4.42 ± 1.90	5.89 ± 2.52	2.57 ± 0.38	3.42 ± 0.50

**Table 3:** IC_20_, IC_50_ and IC_80_ values of BI.

Cell Lines	IC_20_ (μg/mL) ^1^		
	24 h	48 h	72 h
NCI-H1703	1.49 ± 0.08	1.72 ± 0.07	1.83 ± 0.07
SK-LU-1	1.87 ± 0.13	2.91 ± 0.87	3.46 ± 0.72
HLCSC	1.88 ± 0.16	2.65 ± 0.10	2.91 ± 0.21
**Cell Lines**	**IC_50_ (μg/mL) ^2^**		
	**24 h**	**48 h**	**72 h**
NCI-H1703	2.90 ± 0.16	2.93 ± 0.13	2.95 ± 0.11
SK-LU-1	3.96 ± 0.27	4.54 ± 1.35	4.99 ± 1.04
HLCSC	2.88 ± 0.24	2.80 ± 0.11	3.14 ± 0.23
**Cell Lines**	**IC_80_ (μg/mL) ^1^**		
	**24 h**	**48 h**	**72 h**
NCI-H1703	5.65 ± 0.30	5.01 ± 0.22	4.73 ± 0.17
SK-LU-1	8.41 ± 0.57	7.08 ± 2.11	7.19 ± 1.50
HLCSC	4.41 ± 0.37	2.95 ± 0.11	3.38 ± 0.24

^1^ IC_20/80_ values were estimated using the formula ${\mathrm{IC}}_{F}\;=\;(\frac{100-F}{F}{)}^{1/H}\times \;{\mathrm{IC}}_{50}$, where F is the desired percentage of maximal inhibition, i.e., 20% or 80%, and H is hill slope/gradient of the non-linear regression curve. ^2^ IC_50_ data were based on mean values obtained from duplicated dose–response curves in two independent experiments.

**Table 4:** IC_20_, IC_50_ and IC_80_ values of PI.

Cell Lines	IC_20_ (μg/mL) ^1^		
	24 h	48 h	72 h
NCI-H1703	1.74 ± 0.14	1.81 ± 0.14	1.99 ± 0.01
SK-LU-1	1.81 ± 0.00	2.14 ± 0.00	2.03 ± 0.03
HLCSC	3.81 ± 0.15	5.78 ± 0.07	5.89 ± 0.15
**Cell Lines**	**IC_50_ (μg/mL) ^2^**		
	**24 h**	**48 h**	**72 h**
NCI-H1703	2.82 ± 0.22	2.78 ± 0.21	2.92 ± 0.01
SK-LU-1	2.68 ± 0.01	2.69 ± 0.00	2.81 ± 0.04
HLCSC	5.14 ± 0.20	6.10 ± 0.07	6.27 ± 0.16
**Cell Lines**	**IC_80_ (μg/mL) ^1^**		
	**24 h**	**48 h**	**72 h**
NCI-H1703	4.55 ± 0.35	4.26 ± 0.32	4.29 ± 0.02
SK-LU-1	3.96 ± 0.01	3.38 ± 0.00	3.87 ± 0.05
HLCSC	6.94 ± 0.27	6.44 ± 0.07	6.66 ± 0.17

^1^ IC_20/80_ values were estimated using the formula ${\mathrm{IC}}_{F}\;=\;(\frac{100-F}{F}{)}^{1/H}\times \;{\mathrm{IC}}_{50}$, where F is the desired percentage of maximal inhibition, i.e., 20% or 80%, and H is hill slope/gradient of the non-linear regression curve. ^2^ IC_50_ data were based on mean values obtained from duplicated dose–response curves in two independent experiments.

**Table 5:** IC_20_, IC_50_ and IC_80_ values of SAL.

Cell Lines	IC_20_ (μg/mL) ^1^		
	24 h	48 h	72 h
NCI-H1703	0.85 ± 0.31	0.60 ± 0.03	0.54 ± 0.05
SK-LU-1	2.50 ± 0.56	1.22 ± 0.13	1.13 ± 0.04
HLCSC	1.02 ± 0.10	0.71 ± 0.30	0.26 ± 0.04
**Cell Lines**	**IC_50_ (μg/mL) ^2^**		
	**24 h**	**48 h**	**72 h**
NCI-H1703	11.03 ± 4.03	6.00 ± 0.28	4.96 ± 0.42
SK-LU-1	10.40 ± 2.33	9.96 ± 1.10	10.18 ± 0.33
HLCSC	3.46 ± 0.35	4.42 ± 1.90	2.57 ± 0.37
**Cell Lines**	**IC_80_ (μg/mL) ^1^**		
	**24 h**	**48 h**	**72 h**
NCI-H1703	143.92 ± 52.59	60.36 ± 2.78	45.37 ± 3.82
SK-LU-1	43.17 ± 9.66	80.94 ± 8.91	91.89 ± 2.94
HLCSC	11.79 ± 1.20	27.64 ± 11.85	25.32 ± 3.70

^1^ IC_20/80_ values were estimated using the formula ${\mathrm{IC}}_{F}\;=\;(\frac{100-F}{F}{)}^{1/H}\times \;{\mathrm{IC}}_{50}$, where F is the desired percentage of maximal inhibition, i.e., 20% or 80%, and H is hill slope/gradient of the non-linear regression curve. ^2^ IC_50_ data were based on mean values obtained from duplicated dose–response curves in two independent experiments.

## Supplementary Materials

The following are available online, Figure S1: Kinetic dose–response curves of SAL in SK-LU-1 and HLCSC; Figure S2: Kinetic dose–response curves of BI, PI and SAL in NCI-H1703; Figure S3: Representative micrographs of multicellular tumor spheroid model of NCI-H1703 treated with various concentrations of BI and PI; Figure S4: Kinetic dose–response curves of phytochemicals in IMR-90; Figure S5: compilation of kinetic dose–response curves of phytochemicals in SK-LU-1, HLCSC and NCI-H1703; Figure S6: compilation of soft agar micrographs after BI and PI treatments; Spreadsheet S1: Raw data for quantification of cell count with positive nuclear β-catenin after phytochemicals treatments; Spreadsheet S2: Raw data for IC20, IC50 and IC_80_ values of BI, PI and SAL; Spreadsheet S3: dose–response of BI and PI in spheroid vs. monolayer model of NCI-H1703; Spreadsheet S4: Dose–response, migration and invasion of NCI-H1703 after BI and PI treatments.
