# Supplementary figures and images for: Identification of Phytochemical-Based β-Catenin Nuclear Localization Inhibitor in NSCLC: Differential Targeting Population from Member of Isothiocyanates
Source: Molecules. 2021 Jan 13;26(2):399. doi: 10.3390/molecules26020399 (PMC7828655; doi:10.3390/molecules26020399)

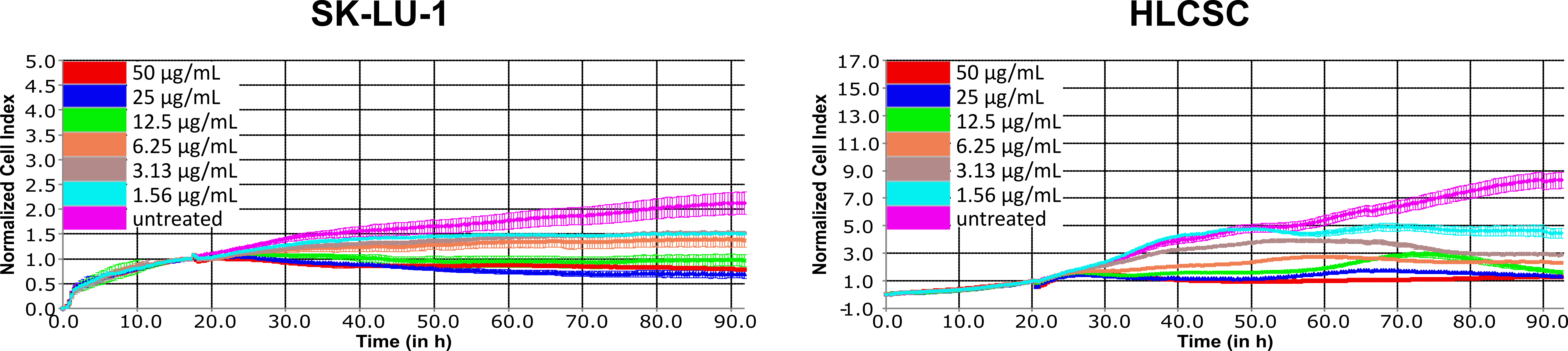

Supplement: Supplementary file 1 [file molecules-26-00399-s001.zip › Figure S1-Kinetic dose–response curves of SAL in SK-LU-1 and HLCSC.png]

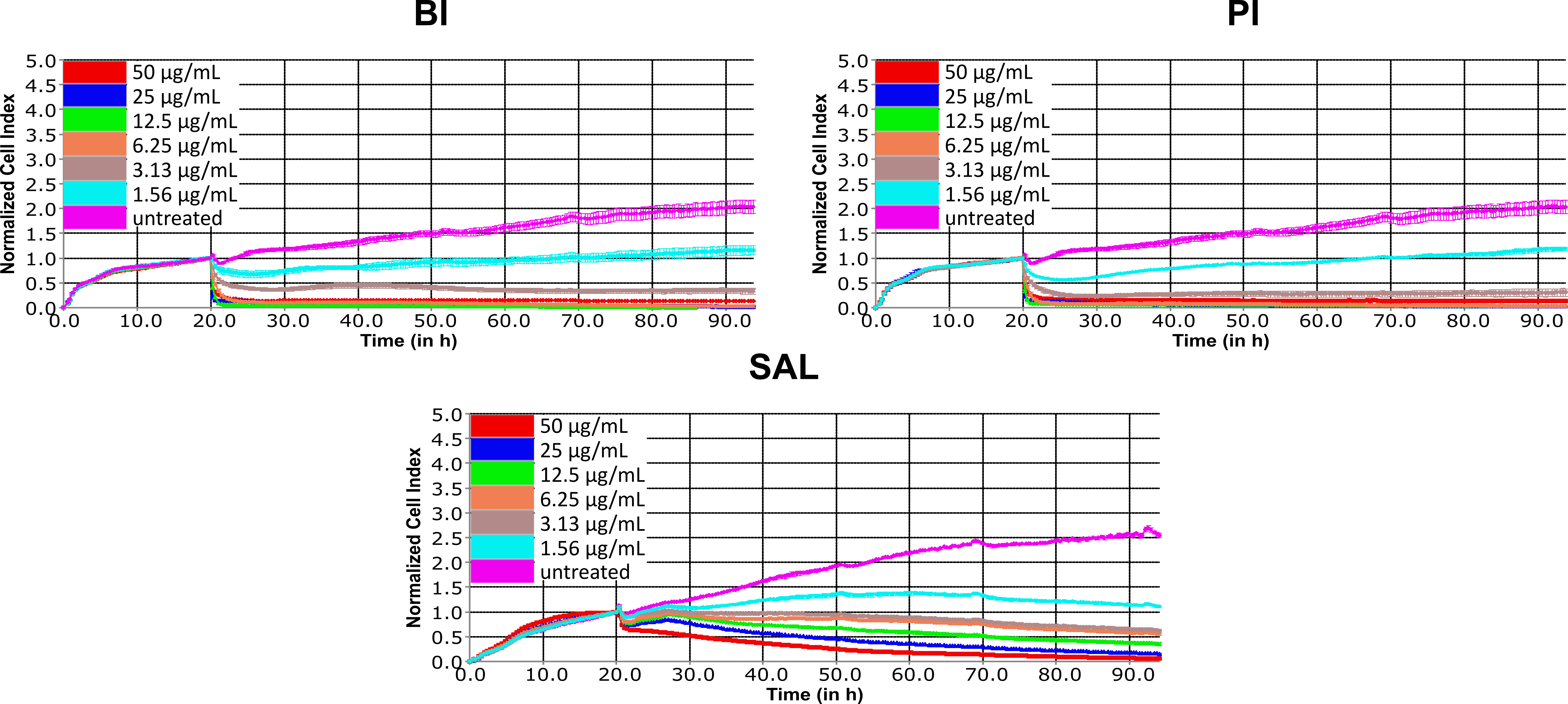

Supplement: Supplementary file 1 [file molecules-26-00399-s001.zip › Figure S2-Kinetic dose–response curves of BI, PI and SAL in NCI-H1703.png]

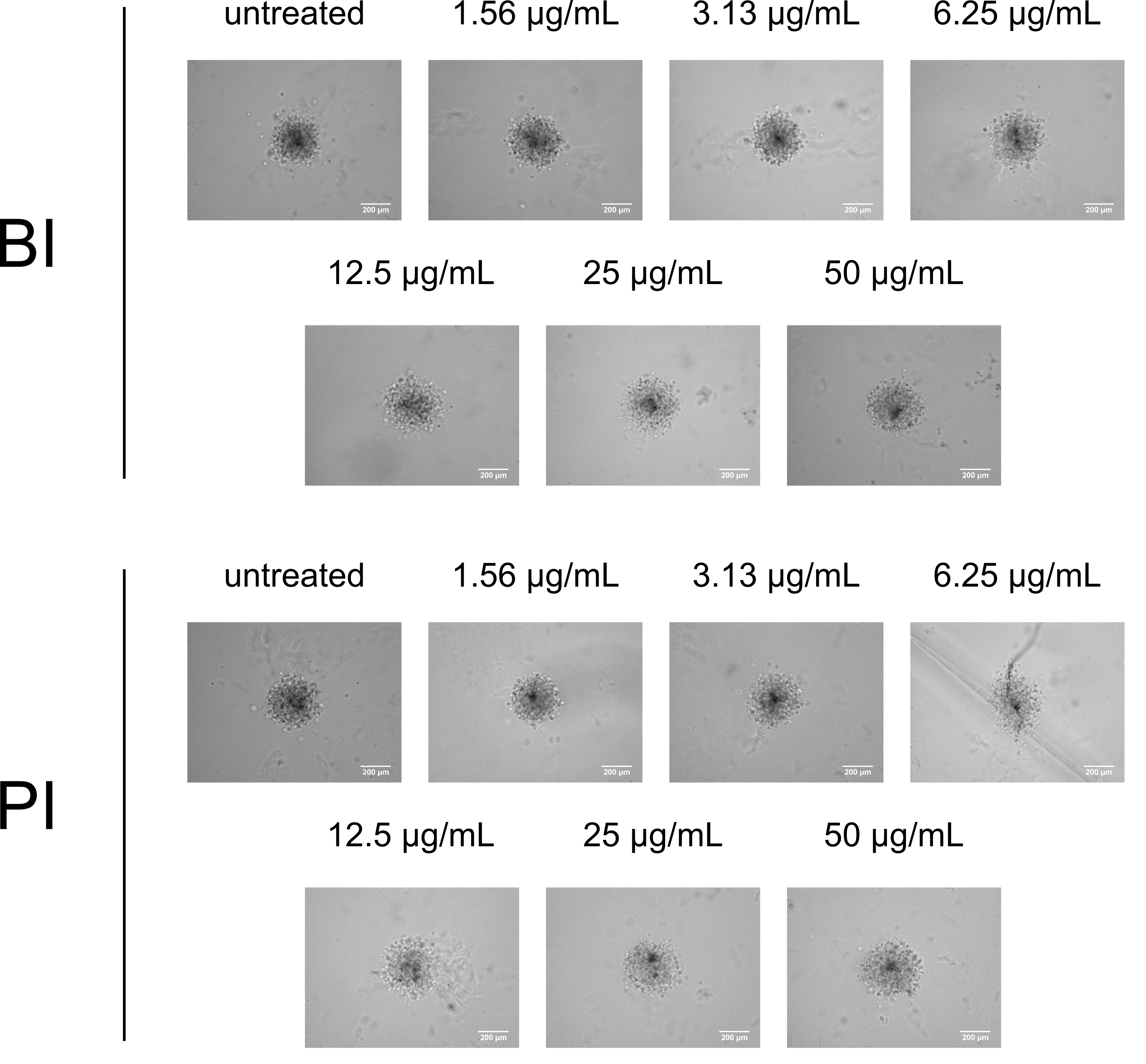

Supplement: Supplementary file 1 [file molecules-26-00399-s001.zip › Figure S3-Representative micrographs of multicellular tumor spheroid model of NCI-H1703 treated with various concentrations of BI and PI.png]

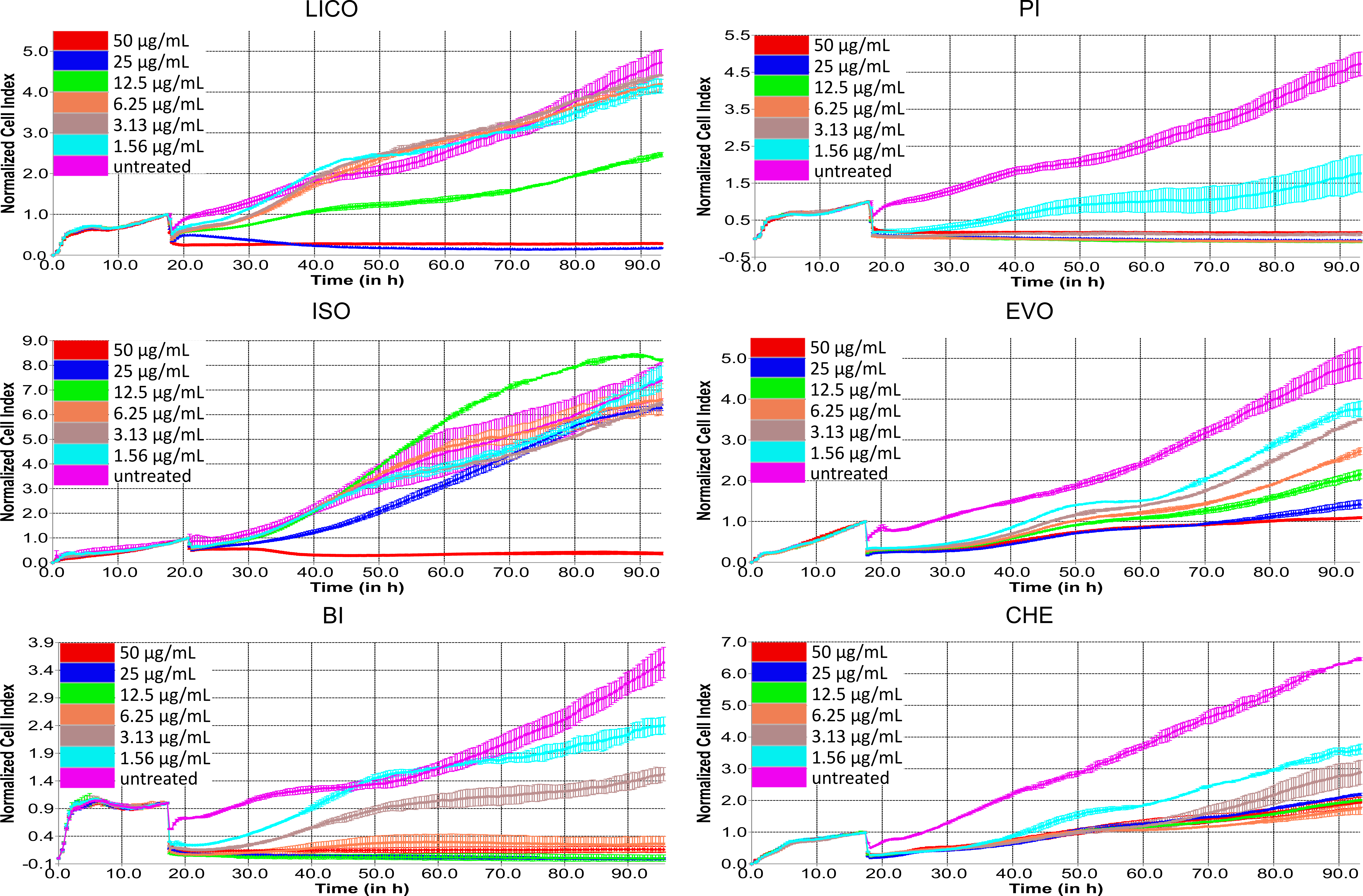

Supplement: Supplementary file 1 [file molecules-26-00399-s001.zip › Figure S4-Kinetic dose–response curves of phytochemicals in IMR-90.png]
